# (3a*R*,8b*R*)-3a,8b-Dihy­droxy-1-(4-meth­oxy­phen­yl)-2-methyl­sulfan­yl-3-nitro-1,8b-di­hydro­indeno­[1,2-*b*]pyrrol-4(3a*H*)-one

**DOI:** 10.1107/S1600536813031279

**Published:** 2013-11-30

**Authors:** R. A. Nagalakshmi, J. Suresh, V. Jeyachandran, R. Ranjith Kumar, P. L. Nilantha Lakshman

**Affiliations:** a Department of Physics, The Madura College, Madurai 625 011, India; b Department of Organic Chemistry, School of Chemistry, Madurai Kamaraj University, Madurai 625 021, India; c Department of Food Science and Technology, University of Ruhuna, Mapalana, Kamburupitiya 81100, Sri Lanka

## Abstract

In the title compound, C_19_H_16_N_2_O_6_S, the pyrrolidine ring adopts a twisted conformation with puckering parameters *q*_2_ = 0.088 (3) Å and Φ_2_ = 61.5 (14)°. The cyclo­pentane ring adopts a twisted conformation with puckering parameters *q*_2_ = 0.099 (2) Å and Φ_2_ = 242.8 (14)°. A weak intra­molecular O—H⋯O inter­action occurs. In the crystal, pairs of C—H⋯O inter­actions generate dimers with graph-set motif *R*_2_^2^(24) and they are interconnected by pairs of O—H⋯O hydrogen bonds, which link the mol­ecules into inversion dimers with graph-set motif *R*_2_^2^(10).

## Related literature

For the importance of pyrrolidine derivatives, see: Cordell (1981 ▶); Morais *et al.* (2009 ▶); Bello *et al.* (2010 ▶); Obniska *et al.* (2010 ▶). For related structures, see: Liu *et al.* (2008 ▶); Ghorbani (2012 ▶). For additional conformation analysis, see: Cremer & Pople (1975 ▶).
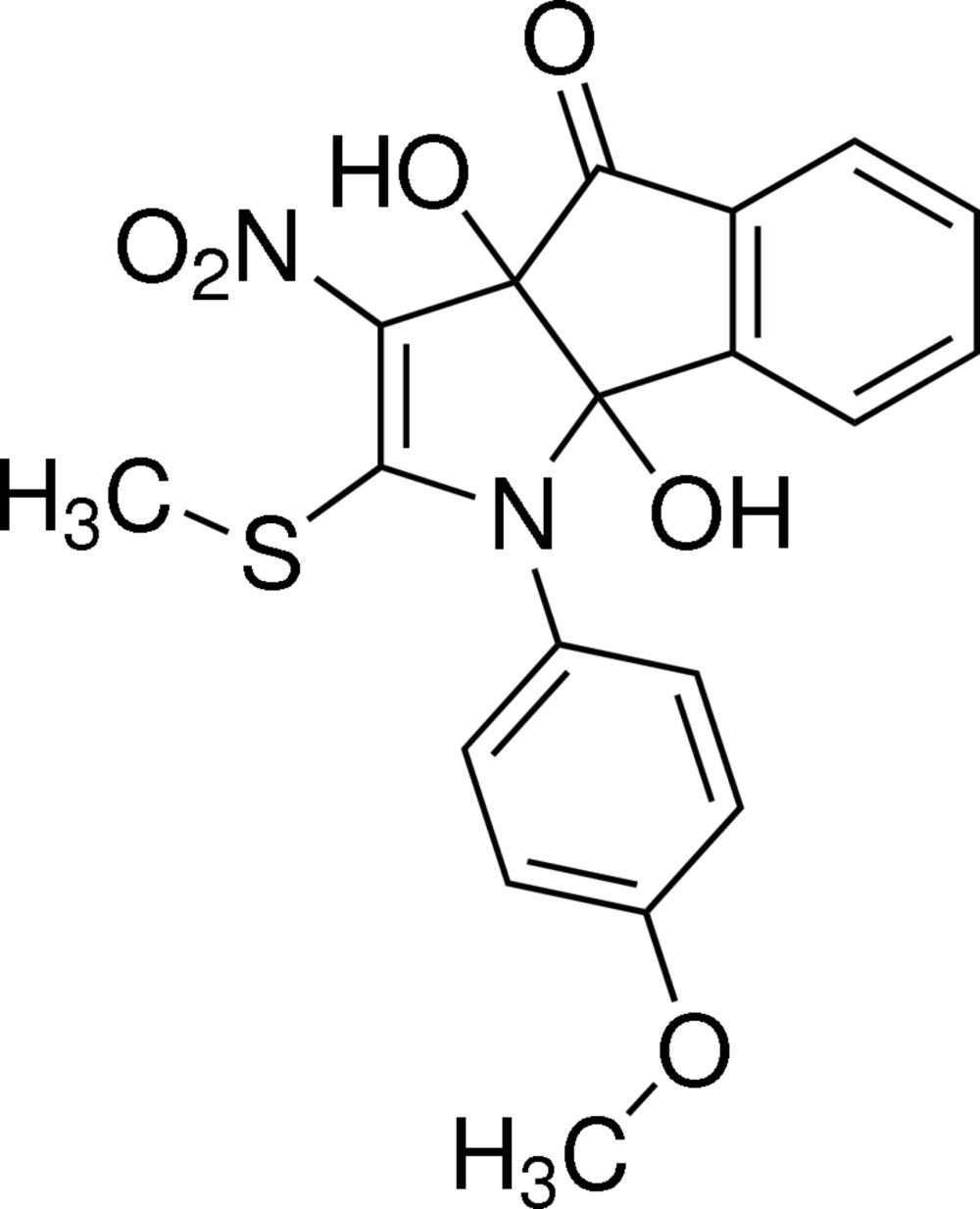


## Experimental

### 

#### Crystal data

C_19_H_16_N_2_O_6_S
*M* *_r_* = 400.40
Monoclinic, 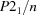

*a* = 13.6601 (7) Å
*b* = 8.5782 (5) Å
*c* = 15.2373 (8) Å
β = 97.684 (3)°
*V* = 1769.46 (17) Å^3^
*Z* = 4
Mo *K*α radiation
μ = 0.23 mm^−1^
*T* = 293 K
0.21 × 0.19 × 0.18 mm

#### Data collection

Bruker Kappa APEXII diffractometer
Absorption correction: multi-scan (*SADABS*; Sheldrick, 1996 ▶) *T*_min_ = 0.967, *T*_max_ = 0.974
13771 measured reflections
3311 independent reflections
2661 reflections with *I* > 2σ(*I*)
*R*_int_ = 0.047

#### Refinement

*R*[*F* ^2^ > 2σ(*F* ^2^)] = 0.053
*wR*(*F* ^2^) = 0.155
*S* = 1.02
3311 reflections
253 parameters
H-atom parameters constrained
Δρ_max_ = 0.42 e Å^−3^
Δρ_min_ = −0.44 e Å^−3^

### 

Data collection: *APEX2* (Bruker, 2004 ▶); cell refinement: *SAINT* (Bruker, 2004 ▶); data reduction: *SAINT*; program(s) used to solve structure: *SHELXS97* (Sheldrick, 2008 ▶); program(s) used to refine structure: *SHELXL97* (Sheldrick, 2008 ▶); molecular graphics: *PLATON* (Spek, 2009 ▶); software used to prepare material for publication: *SHELXL97*.

## Supplementary Material

Crystal structure: contains datablock(s) global, I. DOI: 10.1107/S1600536813031279/zq2211sup1.cif

Structure factors: contains datablock(s) I. DOI: 10.1107/S1600536813031279/zq2211Isup2.hkl

Supplementary material file. DOI: 10.1107/S1600536813031279/zq2211Isup3.cml

Additional supplementary materials:  crystallographic information; 3D view; checkCIF report

## Figures and Tables

**Table 1:** Hydrogen-bond geometry (Å, °)

*D*—H⋯*A*	*D*—H	H⋯*A*	*D*⋯*A*	*D*—H⋯*A*
O3—H3⋯O5	0.82	2.50	3.014 (3)	122
O3—H3⋯O1^i^	0.82	2.06	2.821 (2)	155
C11—H11⋯O6^ii^	0.93	2.60	3.461 (3)	155

Symmetry codes: (i) 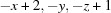; (ii) 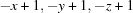.

**Fig. 1.**
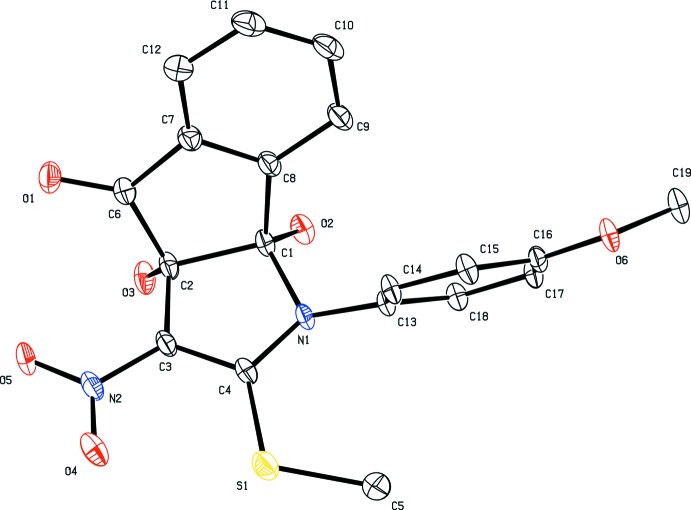
The molecular structure of the title compound, showing 20% probability displacement ellipsoids and the atom-numbering scheme. H-atoms are omitted for clarity.

**Fig. 2.**
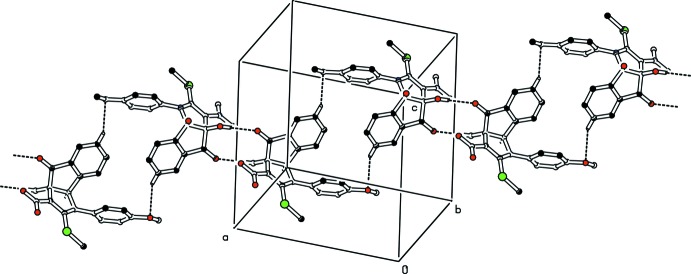
The partial packing diagram showing O—H···O and C—H···O interactions.

## supplementary crystallographic information

### 1. Comment

Pyrrolidine ring system is a frequently encountered structural motif in many biologically relevant alkaloids (Cordell, 1981). Pyrrolidine derivatives possess anticonvulsant (Obniska *et al.*, 2010), anti-angiogenic (Morais *et al.*, 2009) and antitumor activities (Bello *et al.*, 2010). In view of the high medicinal value of these compounds in conjunction with our research interests, prompted us to synthesize and report the X-ray studies of the title compound (Fig. 1).

In the title compound, C_19_H_16_N_2_O_6_S, the pyrrolidine ring (N1/C1/C2/C3/C4) adopts a twisted conformation with the puckering parameters q_2_ = 0.088 (3) Å and Φ_2_ = 61.5 (14) °. The cyclopentane ring (C1/C2/C6–C8) adopts a twisted conformation with the puckering parameters q_2_ = 0.099 (2) Å and Φ_2_ = 242.8 (14) °. The benzene ring of the indane ring is planar with a r.m.s deviation of 0.0113 (1) °. The methoxy benzene ring (C13–C18) is planar with a r.m.s. deviation of 0.0120 Å. The sum of the C—N—C bond angles around N1 atom (352.99 (18)°) is implying a noticeable flattening of the trigonal pyramidal geometry about N1. The conformation of the methylsulfanyl moiety is antiperiplanar, as evidenced from the torsion angles C5—S1—C4—C3 = 165.1 (2) °. The bond length C4—S1 = 1.727 (2) Å is shorter than S1—C5 = 1.804 (3) Å as found in a similar structure (Ghorbani, 2012). The methyl group of the methyl sulfanyl substituent is tilted towards the plane of the benzene ring as indicated by the angle C4—S1—C5 = 106.52 (11) °. Due to the p - π conjugation of atom O6, the bond distance of O6—C16 (1.371 (2) Å) is significantly shorter than the bond distance of O6—C19 (1.427 (3) Å) as found in a similar structure (Liu *et al.*, 2008). The dihedral angle 68.8 (1) ° between the mean planes of the phenyl ring and of the pyrrolidine ring (C1/C3/C4/N1) indicates that the substituent at N1 is in an equatorial position. The shorter bond lengths, C4—N1 (1.352 (3) Å), C3—N2 (1.385 (3) Å) than the normal C—N (1.47 Å) indicate the electron donating effects of nitrogen atoms.

The crystal structure features a weak intramolecular O—H···O interaction and some intermolecular C—H···O and O—H···O interactions. The O3—H3···O1*^i^* (symmetry code: i = -*x*+2, -*y*, -*z*+1) intermolecular interaction connects, inversely related dimers generating a graph set motif *R*_2_^2^(10) (Cremer & Pople, 1975). The C11—H11···O6 intermolecular interaction connects dimers generating a graph set motif *R*_2_^2^(24) (Fig 2).

### 2. Experimental

A mixture of (*E*)-4-methoxy-*N*-(1-(methylthio)-2-nitrovinyl)aniline (1 mmol) with ninhydrin (1 mmol) in presence of glacial AcOH (3–5 drops) was thoroughly ground in a pestle and mortar at room temperature for 2–10 min. The reaction progress was monitored by thin layer chromatography. After completion of the reaction, the reaction mixture was triturated with crushed ice, the resulting solid filtered off and washed with water to afford the pure product. The compound was further recrystallized from ethanol to obtain suitable crystals for X-ray analysis. Melting point 473 K - 475 K. Yield: 95%

### 3. Refinement

H atoms were placed at calculated positions and allowed to ride on their carrier atoms with C—H = 0.93–0.98 Å and O—H = 0.82 Å. *U*_iso_ = 1.2*U*_eq_(C) for CH_2_ and CH groups and *U*_iso_ = 1.5*U*_eq_(C) for CH_3_ and OH groups.

### Crystal data

**Table:**

C_19_H_16_N_2_O_6_S	*F*(000) = 832
*M**_r_* = 400.40	*D*_x_ = 1.503 Mg m^−^^3^
Monoclinic, *P*2_1_/*n*	Mo *K*α radiation, λ = 0.71073 Å
Hall symbol: -p 2yn	Cell parameters from 2000 reflections
*a* = 13.6601 (7) Å	θ = 2–31°
*b* = 8.5782 (5) Å	µ = 0.23 mm^−^^1^
*c* = 15.2373 (8) Å	*T* = 293 K
β = 97.684 (3)°	Block, yellow
*V* = 1769.46 (17) Å^3^	0.21 × 0.19 × 0.18 mm
*Z* = 4	

### Data collection

**Table:**

Bruker Kappa APEXII diffractometer	3311 independent reflections
Radiation source: fine-focus sealed tube	2661 reflections with *I* > 2σ(*I*)
Graphite monochromator	*R*_int_ = 0.047
Detector resolution: 0 pixels mm^-1^	θ_max_ = 25.5°, θ_min_ = 1.9°
ω and φ scans	*h* = −16→15
Absorption correction: multi-scan (*SADABS*; Sheldrick, 1996)	*k* = −10→7
*T*_min_ = 0.967, *T*_max_ = 0.974	*l* = −17→18
13771 measured reflections	

### Refinement

**Table:**

Refinement on *F*^2^	Primary atom site location: structure-invariant direct methods
Least-squares matrix: full	Secondary atom site location: difference Fourier map
*R*[*F*^2^ > 2σ(*F*^2^)] = 0.053	Hydrogen site location: inferred from neighbouring sites
*wR*(*F*^2^) = 0.155	H-atom parameters constrained
*S* = 1.02	*w* = 1/[σ^2^(*F*_o_^2^) + (0.0989*P*)^2^ + 0.7209*P*] where *P* = (*F*_o_^2^ + 2*F*_c_^2^)/3
3311 reflections	(Δ/σ)_max_ < 0.001
253 parameters	Δρ_max_ = 0.42 e Å^−^^3^
0 restraints	Δρ_min_ = −0.44 e Å^−^^3^

### Special details

**Table:**

Geometry. All e.s.d.'s (except the e.s.d. in the dihedral angle between two l.s. planes) are estimated using the full covariance matrix. The cell e.s.d.'s are taken into account individually in the estimation of e.s.d.'s in distances, angles and torsion angles; correlations between e.s.d.'s in cell parameters are only used when they are defined by crystal symmetry. An approximate (isotropic) treatment of cell e.s.d.'s is used for estimating e.s.d.'s involving l.s. planes.
Refinement. Refinement of *F*^2^ against ALL reflections. The weighted *R*-factor *wR* and goodness of fit *S* are based on *F*^2^, conventional *R*-factors *R* are based on *F*, with *F* set to zero for negative *F*^2^. The threshold expression of *F*^2^ > σ (*F*^2^) is used only for calculating *R*-factors(gt) *etc*. and is not relevant to the choice of reflections for refinement. *R*-factors based on *F*.^2^ are statistically about twice as large as those based on *F*, and *R*-factors based on ALL data will be even larger.

### Fractional atomic coordinates and isotropic or equivalent isotropic displacement parameters (Å^2^)

**Table:**

	*x*	*y*	*z*	*U*_iso_*/*U*_eq_	
C1	0.70509 (13)	0.0236 (3)	0.42776 (14)	0.0363 (5)	
C2	0.82032 (13)	0.0315 (3)	0.43000 (14)	0.0375 (5)	
C3	0.82888 (14)	0.0735 (3)	0.33568 (15)	0.0387 (5)	
C4	0.73865 (14)	0.0682 (2)	0.28246 (14)	0.0361 (5)	
C5	0.60238 (18)	0.0213 (4)	0.12776 (17)	0.0575 (7)	
H5A	0.5917	0.0297	0.0644	0.086*	
H5B	0.5551	0.0849	0.1525	0.086*	
H5C	0.5948	−0.0854	0.1446	0.086*	
C6	0.85309 (15)	0.1658 (3)	0.49362 (14)	0.0407 (5)	
C7	0.76369 (15)	0.2479 (3)	0.51268 (14)	0.0410 (5)	
C8	0.67980 (14)	0.1644 (3)	0.48003 (13)	0.0389 (5)	
C9	0.58736 (16)	0.2127 (3)	0.49893 (14)	0.0468 (6)	
H9	0.5307	0.1555	0.4797	0.056*	
C10	0.58276 (19)	0.3479 (3)	0.54700 (16)	0.0557 (7)	
H10	0.5218	0.3826	0.5599	0.067*	
C11	0.6668 (2)	0.4338 (3)	0.57670 (18)	0.0609 (7)	
H11	0.6611	0.5260	0.6077	0.073*	
C12	0.75837 (19)	0.3837 (3)	0.56070 (16)	0.0540 (6)	
H12	0.8151	0.4395	0.5816	0.065*	
C13	0.56455 (13)	0.0879 (2)	0.31000 (13)	0.0337 (5)	
C14	0.54498 (14)	0.2415 (3)	0.28747 (14)	0.0393 (5)	
H14	0.5967	0.3094	0.2813	0.047*	
C15	0.44902 (14)	0.2942 (3)	0.27415 (15)	0.0413 (5)	
H15	0.4355	0.3966	0.2565	0.050*	
C16	0.37246 (13)	0.1939 (3)	0.28725 (14)	0.0367 (5)	
C17	0.39162 (14)	0.0406 (3)	0.30889 (15)	0.0400 (5)	
H17	0.3400	−0.0268	0.3164	0.048*	
C18	0.48858 (14)	−0.0138 (3)	0.31960 (14)	0.0377 (5)	
H18	0.5020	−0.1180	0.3331	0.045*	
C19	0.20231 (15)	0.1754 (3)	0.30760 (19)	0.0563 (7)	
H19A	0.1421	0.2340	0.2963	0.085*	
H19B	0.2181	0.1579	0.3701	0.085*	
H19C	0.1942	0.0771	0.2774	0.085*	
N1	0.66559 (11)	0.0372 (2)	0.33164 (11)	0.0349 (4)	
N2	0.91927 (13)	0.0968 (2)	0.30602 (14)	0.0452 (5)	
O1	0.93701 (11)	0.1960 (2)	0.52411 (12)	0.0554 (5)	
O2	0.67032 (10)	−0.11114 (19)	0.46335 (10)	0.0443 (4)	
H2	0.6844	−0.1868	0.4346	0.066*	
O3	0.86160 (10)	−0.11224 (19)	0.45807 (11)	0.0475 (4)	
H3	0.9208	−0.1119	0.4537	0.071*	
O4	0.92122 (12)	0.1327 (2)	0.22720 (13)	0.0597 (5)	
O5	0.99515 (11)	0.0817 (2)	0.35945 (13)	0.0621 (5)	
O6	0.28043 (9)	0.2606 (2)	0.27626 (11)	0.0473 (4)	
S1	0.72558 (4)	0.08679 (9)	0.16864 (4)	0.0526 (2)	

### Atomic displacement parameters (Å^2^)

**Table:**

	*U*^11^	*U*^22^	*U*^33^	*U*^12^	*U*^13^	*U*^23^
C1	0.0206 (9)	0.0434 (13)	0.0459 (11)	0.0030 (8)	0.0079 (8)	0.0032 (9)
C2	0.0209 (9)	0.0407 (12)	0.0519 (12)	0.0040 (8)	0.0077 (8)	−0.0005 (9)
C3	0.0242 (9)	0.0430 (13)	0.0514 (12)	0.0010 (8)	0.0143 (8)	−0.0048 (9)
C4	0.0274 (9)	0.0350 (11)	0.0483 (11)	0.0048 (8)	0.0138 (8)	0.0023 (9)
C5	0.0478 (13)	0.0747 (19)	0.0499 (13)	0.0045 (12)	0.0060 (10)	−0.0085 (13)
C6	0.0293 (10)	0.0464 (13)	0.0466 (11)	0.0039 (9)	0.0056 (8)	−0.0001 (10)
C7	0.0343 (10)	0.0467 (13)	0.0424 (11)	0.0065 (9)	0.0067 (8)	0.0003 (9)
C8	0.0295 (10)	0.0468 (13)	0.0416 (10)	0.0076 (8)	0.0095 (8)	0.0019 (9)
C9	0.0335 (10)	0.0617 (16)	0.0482 (12)	0.0097 (10)	0.0158 (9)	0.0032 (11)
C10	0.0508 (14)	0.0675 (18)	0.0533 (13)	0.0196 (12)	0.0235 (11)	−0.0003 (12)
C11	0.0675 (17)	0.0617 (18)	0.0571 (15)	0.0136 (13)	0.0216 (13)	−0.0126 (13)
C12	0.0523 (14)	0.0603 (17)	0.0505 (13)	0.0019 (11)	0.0104 (11)	−0.0109 (12)
C13	0.0189 (9)	0.0404 (12)	0.0426 (11)	0.0019 (7)	0.0074 (7)	0.0010 (8)
C14	0.0219 (9)	0.0413 (13)	0.0568 (12)	−0.0010 (8)	0.0136 (8)	0.0046 (10)
C15	0.0272 (10)	0.0400 (13)	0.0583 (13)	0.0042 (8)	0.0118 (9)	0.0090 (10)
C16	0.0200 (9)	0.0449 (13)	0.0457 (11)	0.0024 (8)	0.0068 (7)	−0.0011 (9)
C17	0.0218 (9)	0.0444 (13)	0.0547 (12)	−0.0044 (8)	0.0086 (8)	0.0008 (10)
C18	0.0273 (9)	0.0364 (12)	0.0504 (12)	0.0003 (8)	0.0085 (8)	0.0018 (9)
C19	0.0245 (10)	0.0686 (17)	0.0778 (17)	0.0003 (10)	0.0140 (10)	0.0083 (14)
N1	0.0186 (7)	0.0440 (11)	0.0436 (9)	0.0031 (6)	0.0094 (6)	0.0022 (8)
N2	0.0292 (9)	0.0450 (12)	0.0653 (13)	−0.0016 (7)	0.0203 (8)	−0.0069 (9)
O1	0.0299 (8)	0.0645 (12)	0.0697 (11)	0.0010 (7)	−0.0008 (7)	−0.0141 (9)
O2	0.0334 (8)	0.0465 (10)	0.0547 (9)	0.0002 (6)	0.0126 (6)	0.0093 (7)
O3	0.0247 (7)	0.0448 (10)	0.0719 (11)	0.0070 (6)	0.0022 (7)	0.0016 (8)
O4	0.0439 (9)	0.0645 (12)	0.0767 (12)	−0.0021 (8)	0.0300 (8)	0.0084 (10)
O5	0.0217 (8)	0.0881 (14)	0.0779 (12)	−0.0032 (7)	0.0118 (8)	−0.0144 (10)
O6	0.0194 (6)	0.0518 (10)	0.0723 (10)	0.0048 (6)	0.0117 (6)	0.0072 (8)
S1	0.0402 (3)	0.0725 (5)	0.0474 (4)	0.0029 (3)	0.0146 (3)	0.0101 (3)

### Geometric parameters (Å, º)

**Table:**

C1—O2	1.387 (3)	C11—C12	1.375 (4)
C1—N1	1.496 (3)	C11—H11	0.9300
C1—C8	1.512 (3)	C12—H12	0.9300
C1—C2	1.571 (2)	C13—C14	1.378 (3)
C2—O3	1.399 (3)	C13—C18	1.379 (3)
C2—C3	1.501 (3)	C13—N1	1.443 (2)
C2—C6	1.533 (3)	C14—C15	1.376 (3)
C3—C4	1.383 (3)	C14—H14	0.9300
C3—N2	1.385 (3)	C15—C16	1.389 (3)
C4—N1	1.352 (3)	C15—H15	0.9300
C4—S1	1.727 (2)	C16—O6	1.371 (2)
C5—S1	1.804 (3)	C16—C17	1.372 (3)
C5—H5A	0.9600	C17—C18	1.393 (3)
C5—H5B	0.9600	C17—H17	0.9300
C5—H5C	0.9600	C18—H18	0.9300
C6—O1	1.206 (3)	C19—O6	1.427 (3)
C6—C7	1.472 (3)	C19—H19A	0.9600
C7—C12	1.383 (3)	C19—H19B	0.9600
C7—C8	1.386 (3)	C19—H19C	0.9600
C8—C9	1.395 (3)	N2—O5	1.236 (3)
C9—C10	1.378 (4)	N2—O4	1.244 (3)
C9—H9	0.9300	O2—H2	0.8200
C10—C11	1.388 (4)	O3—H3	0.8200
C10—H10	0.9300		
			
O2—C1—N1	110.51 (17)	C12—C11—H11	119.7
O2—C1—C8	110.19 (16)	C10—C11—H11	119.7
N1—C1—C8	112.04 (17)	C11—C12—C7	118.1 (2)
O2—C1—C2	114.95 (16)	C11—C12—H12	121.0
N1—C1—C2	104.30 (15)	C7—C12—H12	121.0
C8—C1—C2	104.65 (16)	C14—C13—C18	120.52 (18)
O3—C2—C3	115.10 (17)	C14—C13—N1	119.52 (17)
O3—C2—C6	113.41 (17)	C18—C13—N1	119.71 (19)
C3—C2—C6	111.82 (18)	C15—C14—C13	120.01 (19)
O3—C2—C1	109.29 (17)	C15—C14—H14	120.0
C3—C2—C1	101.28 (15)	C13—C14—H14	120.0
C6—C2—C1	104.66 (16)	C14—C15—C16	119.7 (2)
C4—C3—N2	125.2 (2)	C14—C15—H15	120.1
C4—C3—C2	112.11 (17)	C16—C15—H15	120.1
N2—C3—C2	122.27 (18)	O6—C16—C17	124.89 (18)
N1—C4—C3	110.34 (18)	O6—C16—C15	114.77 (19)
N1—C4—S1	126.15 (16)	C17—C16—C15	120.34 (18)
C3—C4—S1	123.39 (15)	C16—C17—C18	119.81 (19)
S1—C5—H5A	109.5	C16—C17—H17	120.1
S1—C5—H5B	109.5	C18—C17—H17	120.1
H5A—C5—H5B	109.5	C13—C18—C17	119.5 (2)
S1—C5—H5C	109.5	C13—C18—H18	120.2
H5A—C5—H5C	109.5	C17—C18—H18	120.2
H5B—C5—H5C	109.5	O6—C19—H19A	109.5
O1—C6—C7	126.3 (2)	O6—C19—H19B	109.5
O1—C6—C2	125.94 (19)	H19A—C19—H19B	109.5
C7—C6—C2	107.72 (17)	O6—C19—H19C	109.5
C12—C7—C8	121.7 (2)	H19A—C19—H19C	109.5
C12—C7—C6	127.7 (2)	H19B—C19—H19C	109.5
C8—C7—C6	110.5 (2)	C4—N1—C13	124.66 (17)
C7—C8—C9	120.0 (2)	C4—N1—C1	111.19 (15)
C7—C8—C1	111.45 (17)	C13—N1—C1	117.14 (15)
C9—C8—C1	128.5 (2)	O5—N2—O4	122.52 (18)
C10—C9—C8	117.8 (2)	O5—N2—C3	118.4 (2)
C10—C9—H9	121.1	O4—N2—C3	119.08 (19)
C8—C9—H9	121.1	C1—O2—H2	109.5
C9—C10—C11	121.7 (2)	C2—O3—H3	109.5
C9—C10—H10	119.1	C16—O6—C19	117.35 (18)
C11—C10—H10	119.1	C4—S1—C5	106.52 (11)
C12—C11—C10	120.6 (3)		
			
O2—C1—C2—O3	7.7 (2)	C7—C8—C9—C10	2.8 (3)
N1—C1—C2—O3	−113.49 (18)	C1—C8—C9—C10	−178.9 (2)
C8—C1—C2—O3	128.67 (18)	C8—C9—C10—C11	−0.5 (4)
O2—C1—C2—C3	129.54 (19)	C9—C10—C11—C12	−1.6 (4)
N1—C1—C2—C3	8.4 (2)	C10—C11—C12—C7	1.4 (4)
C8—C1—C2—C3	−109.45 (18)	C8—C7—C12—C11	0.9 (4)
O2—C1—C2—C6	−114.1 (2)	C6—C7—C12—C11	−174.7 (2)
N1—C1—C2—C6	124.73 (18)	C18—C13—C14—C15	−0.2 (3)
C8—C1—C2—C6	6.9 (2)	N1—C13—C14—C15	−174.4 (2)
O3—C2—C3—C4	109.7 (2)	C13—C14—C15—C16	2.8 (3)
C6—C2—C3—C4	−119.03 (19)	C14—C15—C16—O6	177.0 (2)
C1—C2—C3—C4	−8.1 (2)	C14—C15—C16—C17	−3.4 (3)
O3—C2—C3—N2	−63.6 (3)	O6—C16—C17—C18	−179.1 (2)
C6—C2—C3—N2	67.7 (3)	C15—C16—C17—C18	1.4 (3)
C1—C2—C3—N2	178.6 (2)	C14—C13—C18—C17	−1.9 (3)
N2—C3—C4—N1	177.5 (2)	N1—C13—C18—C17	172.37 (19)
C2—C3—C4—N1	4.4 (3)	C16—C17—C18—C13	1.3 (3)
N2—C3—C4—S1	1.3 (3)	C3—C4—N1—C13	151.4 (2)
C2—C3—C4—S1	−171.80 (15)	S1—C4—N1—C13	−32.5 (3)
O3—C2—C6—O1	49.2 (3)	C3—C4—N1—C1	1.8 (2)
C3—C2—C6—O1	−82.9 (3)	S1—C4—N1—C1	177.84 (15)
C1—C2—C6—O1	168.3 (2)	C14—C13—N1—C4	−48.6 (3)
O3—C2—C6—C7	−129.12 (18)	C18—C13—N1—C4	137.1 (2)
C3—C2—C6—C7	98.72 (19)	C14—C13—N1—C1	99.4 (2)
C1—C2—C6—C7	−10.1 (2)	C18—C13—N1—C1	−74.9 (3)
O1—C6—C7—C12	7.6 (4)	O2—C1—N1—C4	−130.78 (17)
C2—C6—C7—C12	−174.1 (2)	C8—C1—N1—C4	105.92 (19)
O1—C6—C7—C8	−168.5 (2)	C2—C1—N1—C4	−6.7 (2)
C2—C6—C7—C8	9.9 (3)	O2—C1—N1—C13	77.1 (2)
C12—C7—C8—C9	−3.1 (3)	C8—C1—N1—C13	−46.2 (2)
C6—C7—C8—C9	173.2 (2)	C2—C1—N1—C13	−158.86 (17)
C12—C7—C8—C1	178.3 (2)	C4—C3—N2—O5	−172.1 (2)
C6—C7—C8—C1	−5.3 (3)	C2—C3—N2—O5	0.3 (3)
O2—C1—C8—C7	122.85 (19)	C4—C3—N2—O4	8.2 (3)
N1—C1—C8—C7	−113.67 (19)	C2—C3—N2—O4	−179.4 (2)
C2—C1—C8—C7	−1.3 (2)	C17—C16—O6—C19	14.1 (3)
O2—C1—C8—C9	−55.6 (3)	C15—C16—O6—C19	−166.3 (2)
N1—C1—C8—C9	67.9 (3)	N1—C4—S1—C5	−10.5 (2)
C2—C1—C8—C9	−179.7 (2)	C3—C4—S1—C5	165.1 (2)

### Hydrogen-bond geometry (Å, º)

**Table:**

*D*—H···*A*	*D*—H	H···*A*	*D*···*A*	*D*—H···*A*
O3—H3···O5	0.82	2.50	3.014 (3)	122
O3—H3···O1^i^	0.82	2.06	2.821 (2)	155
C11—H11···O6^ii^	0.93	2.60	3.461 (3)	155

Symmetry codes: (i) −*x*+2, −*y*, −*z*+1; (ii) −*x*+1, −*y*+1, −*z*+1.
